# Lipid Polyunsaturated Fatty Acid Chains in Mouse Kidneys Were Increased within 5 min of a Single High Dose Whole Body Irradiation

**DOI:** 10.3390/ijms241512439

**Published:** 2023-08-04

**Authors:** Wenxin Li, Chi Zhang, Shuhei Aramaki, Lili Xu, Shogo Tsuge, Takumi Sakamoto, Md. Al Mamun, Ariful Islam, Takamitsu Hayakawa, Yusuke Takanashi, Maxime Dubail, Kenta Konishi, Tomohito Sato, Tomoaki Kahyo, Charles Fouillade, Katsumasa Nakamura, Mitsutoshi Setou

**Affiliations:** 1 Department of Radiation Oncology, Hamamatsu University School of Medicine, Handayama 1-20-1, Higashi-ku, Hamamatsu 431-3192, Shizuoka, Japan; d20101@hama-med.ac.jp (W.L.);; 2 Department of Cellular and Molecular Anatomy, Hamamatsu University School of Medicine, 1-20-1 Handayama, Higashi-ku, Hamamatsu 431-3192, Shizuoka, Japan; zhangchi07.pegasus@gmail.com (C.Z.); setou@hama-med.ac.jp (M.S.); 3 Department of Systems Molecular Anatomy, Institute for Medical Photonics Research, Preeminent Medical Photonics Education & Research Center Hamamatsu, Hamamatsu 431-3192, Shizuoka, Japan; 4 International Mass Imaging Center, Hamamatsu University School of Medicine, 1-20-1 Handayama, Higashi-Ku, Hamamatsu 431-3192, Shizuoka, Japan; 5 First Department of Surgery, Hamamatsu University School of Medicine, 1-20-1 Handayama, Higashi-ku, Hamamatsu 431-3192, Shizuoka, Japan; 6 Institut Curie, Centre de Recherche, INSERM U612, 91405 Orsay, France

**Keywords:** polyunsaturated fatty acid, lipidomic, high dose radiation, kidney

## Abstract

To understand the ultra-early reaction of normal organ lipids during irradiation, we investigated the response of lipids, including polyunsaturated fatty acid (PUFA) chains, which are particularly susceptible to damage by ROS, in mice’s kidneys, lungs, brains, and livers within 5 min of single high-dose irradiation. In this study, we set up three groups of C56BL/6 male mice and conducted whole-body irradiation with 0 Gy, 10 Gy, and 20 Gy single doses. Kidney, lung, brain, and liver tissues were collected within 5 min of irradiation. PUFA-targeted and whole lipidomic analyses were conducted using liquid chromatography–tandem mass spectrometry (LC-MS/MS). The results showed that PUFA chains of kidney phosphatidylcholine (PC), phosphatidylethanolamine (PE), and triacylglycerol (TG) significantly increased within 5 min of 10 Gy and 20 Gy irradiation. The main components of increased PUFA chains in PC and PE were C18:2, C20:4, and C22:6, and in TG the main component was C18:2. The kidney lipidomes also showed significant changes from the perspective of lipid species, mainly dominated by an increase in PC, PE, TG, and signal lipids, while lipidomes of the lung, brain, and liver were slightly changed. Our results revealed that acute PUFA chains increase and other lipidomic changes in the kidney upon whole-body irradiation within 5 min of irradiation. The significantly increased lipids also showed a consistent preference for possessing PUFA chains. The lipidomic changes varied from organ to organ, which indicates that the response upon irradiation within a short time is tissue-specific.

## 1. Introduction

Radiotherapy is a standard treatment modality for cancer patients, aiming to destroy tumor tissue while minimizing damage to surrounding healthy tissue [1]. Fractionated external beam radiation therapy, delivering 1.8–2.0 Gy per fraction over several weeks, is commonly employed. However, with advances in clinical radiation oncology techniques, stereotactic body radiation therapy (SBRT) has become widely used in clinical practice (8–30 Gy per fraction) [2]. It has been the standard treatment for early-stage lung cancer, and more and more experience has been gained in the treatment of primary pancreatic, liver, kidney, prostate and breast cancers. SBRT is particularly suitable for the treatment of limited metastatic tumors, especially brain, lung, liver and spinal metastases [3]. Although the irradiation target of these techniques is focused as much as possible on the tumor area, some normal tissue remains in the high-dose area. According to general principles, the small number of large dose fractions is more effective at killing healthy cells than tumor cells [4]. Ionizing radiation exerts its effects by generating reactive oxygen species (ROS) through energy transfer to water molecules, resulting in oxidative stress and direct ionization of DNA. With the rapid advances in mass spectrometry, the identification and quantitative assessment of lipids have been facilitated [5,6], and the effect of ROS on lipids has been revealed. Lipids play vital roles in cellular membranes and various cellular processes, and studies have demonstrated that ionizing radiation can modulate lipid profiles in several types of cancer [7].

Polyunsaturated fatty acids (PUFAs), a component of lipids, are particularly vulnerable to ROS-induced damage due to their high degree of double bonds, which can lead to lipid peroxidation [8]. Studies have shown that ROS seize hydrogen atoms from polyunsaturated and monounsaturated fatty acids tens to tens of thousands of times faster than that of saturated fatty acids [9]. Lipid peroxidation has been implicated in mediating inflammatory reactions and triggering programmed cell death, such as ferroptosis [10,11,12]. Previous investigations have revealed a decrease in PUFA chains within 72 h of irradiation [13]. It has been reported that the PUFA chains of phospholipids are relatively reduced in comparison to prostate cancer and its surrounding tissues [14]. Moreover, recent studies have emphasized the significance of phospholipids containing long PUFA chains in regulating cellular survival, particularly in driving cells toward ferroptosis [15]. However, existing research has primarily focused on lipid changes in the days to weeks following irradiation, with very little known about the ultra-early lipid changes in vivo post-irradiation.

Considering the rapid metabolism of lipids, our study aimed to investigate early-stage lipid changes. Based on previous findings, we hypothesized that PUFA chains may exhibit a decreasing trend immediately after irradiation. To explore this hypothesis, we conducted a comprehensive analysis of PUFA-targeted whole lipidomics using liquid chromatography–tandem mass spectrometry (LC-MS/MS) on kidney, lung, brain, and liver tissues obtained from mice within five minutes of single high-dose whole-body irradiation.

## 2. Results

### 2.1. Within 5 min of Irradiation, the PUFA Chain of Kidney Lipids Significantly Increased

**Figure 1:**
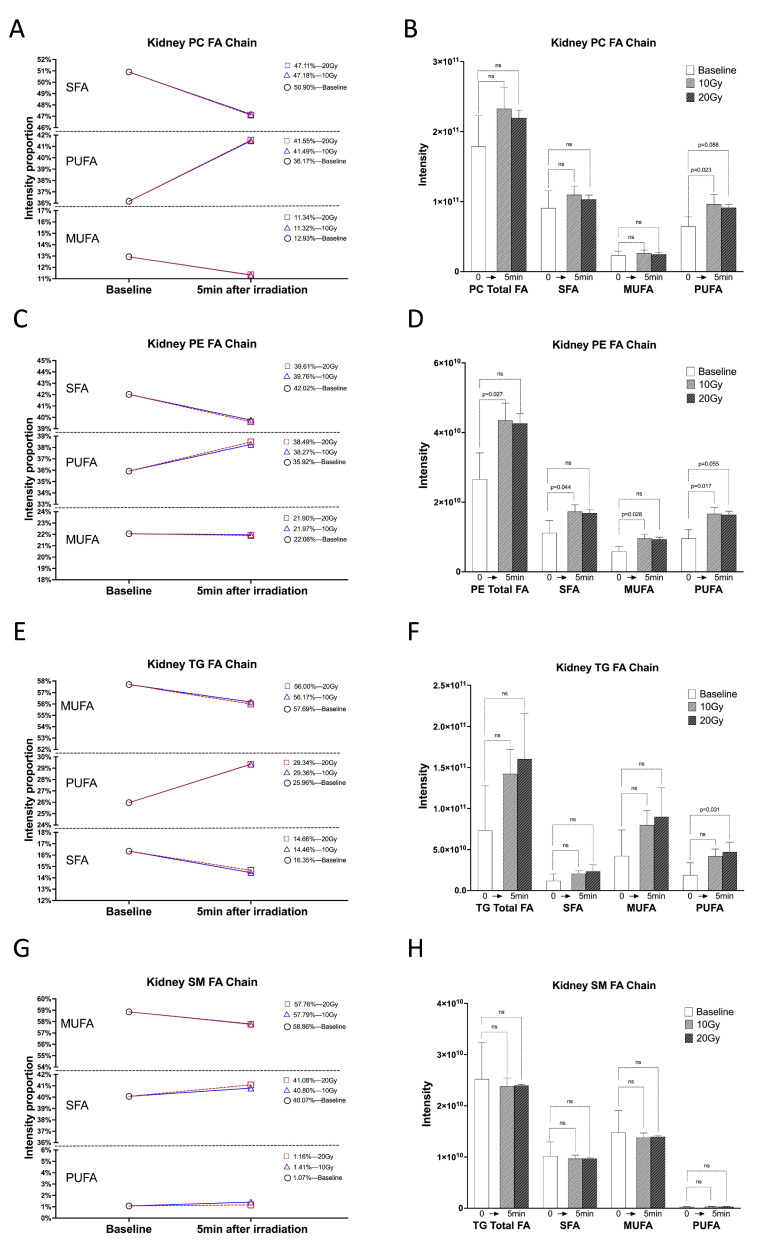
PUFA chains of kidney lipid increased within 5 min of 10 Gy and 20 Gy irradiation. (**A**,**C**,**E**,**G**) Proportional changes in SFA, MUFA, and PUFA chains intensities of kidney PC, PE, TG, and SM under 10 Gy and 20 Gy irradiation compared to 0 Gy. (**B**,**D**,**F**,**H**) Intensity histograms of total FA, SFA, MUFA, and PUFA chains in kidney PC, PE, TG, and SM under 0 Gy, 10 Gy, and 20 Gy irradiation. ns: no significant.

Within 5 min of irradiation, we observed a significant increase in PUFA chains in kidney PC, PE, and TG. The proportion of PUFA chains in kidney PC increased by 5.33% and 5.39% after 10 and 20 Gy irradiation, respectively. Concurrently, the proportion of MUFA chains in kidney PC decreased by 1.61% and 1.59% under 10 and 20 Gy irradiation, respectively. Additionally, the proportion of SFA chains in kidney PC decreased by 3.72% and 3.79% under 10 Gy and 20 Gy irradiation, respectively (Figure 1A). While there were no significant changes observed in the total amount of FA chains, total SFA chains, and total MUFA chains within 5 min of irradiation in PC, the total PUFA chains increased significantly by 49.41% (*p* = 0.023) after 10 Gy irradiation and by 41.39% (*p* = 0.088) after 20 Gy irradiation (Figure 1B) (Table S2).

Similarly, the proportion of PUFA chains in kidney PE increased by 2.36% and 2.58% after 10 and 20 Gy irradiation, respectively. Conversely, the proportion of MUFA chains in kidney PE decreased by 0.09% and 0.16% under 10 Gy and 20 Gy irradiation, while the proportion of SFA chains in kidney PE decreased by 2.27% and 2.42% (Figure 1C). In terms of quantitative analysis, the total amount of FA chains in PE increased by 63.50% after 10 Gy irradiation (*p* = 0.027). Specifically, the amounts of total SFA chains, total MUFA chains, and total PUFA chains in PE increased by 54.67% (*p* = 0.044), 62.84% (*p* = 0.028), and 74.21% (*p* = 0.017), respectively. Following 20 Gy irradiation, the amounts of total SFA chains, total MUFA chains, and total PUFA chains increased by 50.68% (*p* = 0.145), 58.72% (*p* = 0.088), and 71.34% (*p* = 0.055), respectively (Figure 1D) (Table S2).

Regarding kidney TG, the proportion of PUFA chains increased by 3.41% and 3.38% after 10 and 20 Gy irradiation, respectively. Additionally, the proportion of MUFA chains in kidney TG decreased by 1.52% and 1.69%, and the proportion of SFA chains decreased by 1.89% and 1.69% under 10 Gy and 20 Gy irradiation, respectively (Figure 1E). Quantitatively, the total amount of FA chains, SFA chains, and MUFA chains did not exhibit significant changes. However, the total amount of PUFA chains increased significantly by 148.19% (*p* = 0.031) within 5 min of 20 Gy irradiation (Figure 1F).

We also examined the changes in FA chains in kidney sphingomyelin (SM) and found that the proportion of PUFA chains in kidney SM increased by 0.34% and 0.12% after 10 Gy and 20 Gy irradiation, respectively. The proportion of MUFA chains in kidney SM decreased by 1.07% and 1.1% under 10 Gy and 20 Gy irradiation, respectively. Moreover, the proportion of SFA chains in kidney TG increased by 0.1% and 1.01% at 10 Gy and 20 Gy irradiation, respectively (Figure 1G). However, there were no significant changes in the total amount of FA chains or the total amounts of PUFA, MUFA, and SFA chains in SM (Figure 1H) (Table S2).

Finally, we calculated the proportion of each lipid class in the whole lipids of the kidney and all four lipid classes—PC, PE, TG, and SM—each of which accounted for more than 5% of the total lipids in the kidney. We considered them as the major lipid classes (Figure S1). Based on the above results, it is evident that the major kidney lipid classes, including PC, PE, and TG, exhibit a significant increase in both the proportion and intensity of PUFA chains within 5 min of irradiation.

### 2.2. The C18:2, C20:4, and C22:6 FA Chains of Kidney Lipids Were Significantly Increased within 5 min of Irradiation

**Figure 2:**
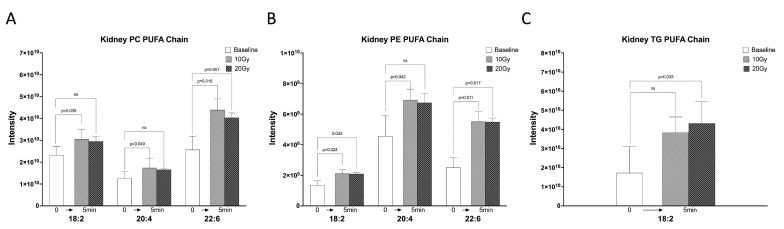
The 18:2, C20:4, and C22:6 chains as the main PUFA chains increased significantly within 5 min of irradiation. (**A**) Intensity histograms of PUFA chains (C18:2, C20:4, and C22:6) in kidney PC under 0 Gy, 10 Gy, and 20 Gy irradiation. (**B**) Intensity histograms of PUFA chains (C18:2, C20:4, and C22:6) in kidney PE under 0 Gy, 10 Gy, and 20 Gy irradiation. (**C**) Intensity histograms of PUFA chains (C18:2) in kidney TG under 0 Gy, 10 Gy, and 20 Gy irradiation. ns: no significant.

We examined the specific changes in PUFA chains within 5 min of radiation. Our findings revealed that the intensity of the C18:2, C20:4, and C22:6 FA chains accounted for a significant proportion of the total FA chains in kidney PC. Specifically, C18:2 chain intensity accounted for 35.96%, C20:4 chain intensity accounted for 19.46%, and C22:6 chain intensity accounted for 39.76% of the total FA chains in PC. In comparison, the number of these FA chains accounted for 22.45%, 19.39%, and 20.41% of the total amount of FA chains in PC without irradiation. Following 10 Gy and 20 Gy irradiation, the intensity of the C18:2 chain in PC increased by 31.21% (*p* = 0.039) and 26.9% (*p* = 0.161), the C20:4 chain increased by 38.40% (*p* = 0.049) and 31.52% (*p* = 0.181), and the C22:6 chain increased by 71.03% (*p* = 0.016) and 57.27% (*p* = 0.051) (Figure 2A).

In PE, we found that the intensity of C18:2, C20:4, and C22:6 accounted for 14.21%, 47.75%, and 26.34% of the total FA chains, and the number of these FA chains accounted for 24.11%, 22.32%, and 23.21% of the total amount of FA chains without irradiation. The intensity of the C18:2 chain in PE increased by 56.37% (*p* = 0.024) and 53.79% (*p* = 0.033), the C20:4 chain increased by 51.75% (*p* = 0.042) and 47.74% (*p* = 0.158), and the C22:6 chain increased by 118.72% (*p* = 0.011) and 118.08% (*p* = 0.017), respectively, within 5 min of 10 Gy and 20 Gy radiation (Figure 2B).

Interestingly, in TG, the intensity of the C18:2 FA chains accounted for 91.02% of the total FA chains of TG, while the amount of C18:2 chains accounted for 43.20% of the FA chains of TG without irradiation. The amount of C18:2 chains in TG increased by 121.18% (*p* = 0.238) and 125.01% (*p* = 0.033) within 5 min of 10 Gy and 20 Gy irradiation, respectively (Figure 2C).

### 2.3. Significant Increase in Kidney Lipidomes within 5 min of Irradiation

**Figure 3:**
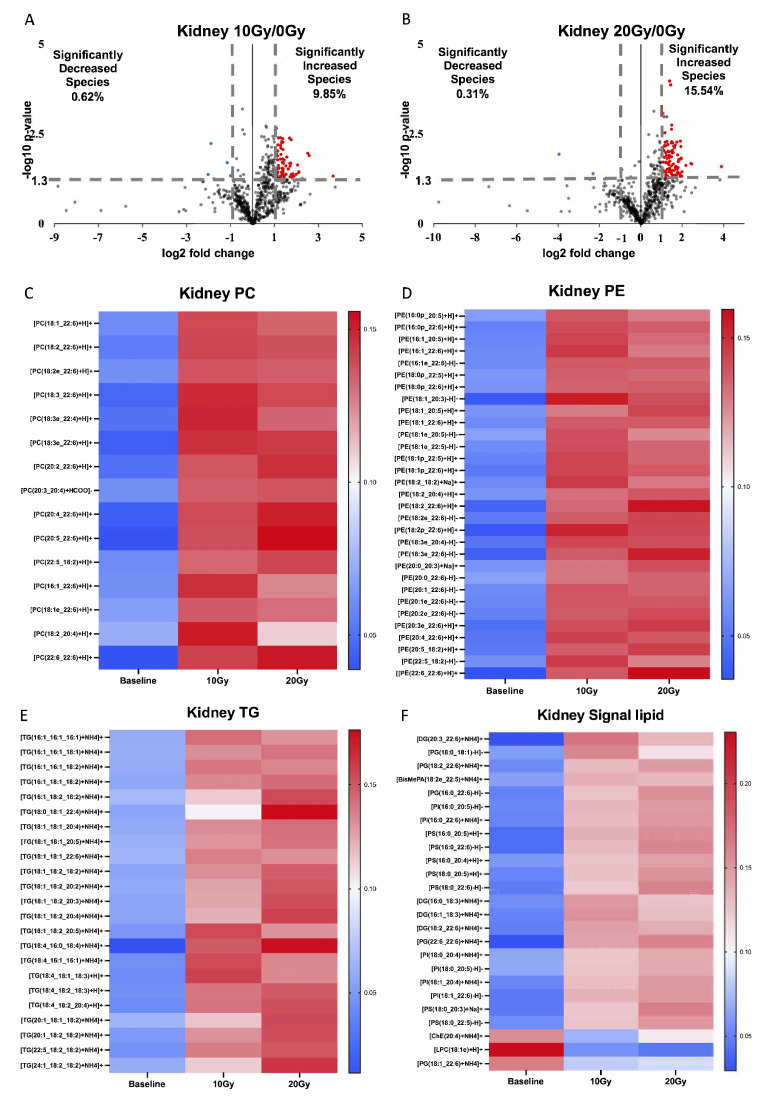
Kidney lipidome altered within 5 min of irradiation. (**A**) Volcano plot of lipid profile under comparison between 10 Gy and 0 Gy irradiation. (**B**) Volcano plot of lipid profile under comparison between 20 Gy and 0 Gy irradiation. (**C**) All significantly increased PC species upon 10 Gy and 20 Gy irradiation. (**D**) All significantly increased PE species upon 10 Gy and 20 Gy irradiation. (**E**) All significantly increased TG species upon 10 Gy and 20 Gy irradiation. (**F**) All other significantly increased lipid species upon 10 Gy and 20 Gy irradiation. The vertical line represents a change of more than 2-fold. The horizontal line represents *p*-value ≤ 0.05. Blue dots: significant decrease in lipid species (FC < 0.5, *p*-value ≤ 0.05). Red dots: significant increase in lipid species (FC > 2, *p*-value ≤ 0.05).

We further investigated the overall changes in the kidney lipid profile within 5 min of irradiation. Using volcano plots based on lipidomic data, we identified significantly altered lipid species with *p*-values less than or equal to 0.05 and multiplicative changes higher than 2-fold (Table S1). Among the kidney lipid group, 9.85% of the lipids showed a significant increase. In comparison, 0.62% showed a significant decrease after 10 Gy irradiation (Figure 3A). Similarly, after 20 Gy irradiation, 15.54% of the lipids showed a significant increase. In comparison, 0.31% showed a significant decrease (Figure 3B). These results indicate that the kidney lipidomes exhibited significant changes within a short time after radiation, primarily characterized by an increase in lipids, with less than 1% of the lipid species showing a decrease after irradiation at both doses.

Furthermore, we classified the significantly changed lipid species into four categories: PC, PE, TG, and signaling lipids. Interestingly, all 15 changed PC species, 31 changed PE species, and 23 changed TG species exhibited significant increases (Figure 3C–E), consistent with the changes observed in PUFA chains. Among the altered signaling lipids, three species showed a significant decrease (Figure 3F). We also investigated the composition of FA chains in the significantly increased lipids. Among the 11 significantly changed PCs under 10 Gy irradiation, 20 chains were PUFA chains, and 2 were MUFA chains without any SFA chains. Similarly, among the 11 significantly changed PCs under 20 Gy irradiation, 21 chains were PUFA chains, and 1 was a MUFA chain, again without any SFA chains. Among the significantly changed PEs, 25 PEs with 50 FA chains showed significant changes after 10 Gy irradiation. Among these FA chains, 35 were PUFA chains, 10 were MUFA chains, and 5 were SFA chains. After 20 Gy irradiation, 27 PEs with 54 FA chains exhibited significant changes, including 37 PUFA chains, 11 MUFA chains, and 6 SFA chains. Regarding the TG species, 1 TG with significant changes after 10 Gy irradiation had 2 PUFA chains and 1 MUFA chain without any SFA chains. However, after 20 Gy irradiation, 23 TG species (containing 69 FA chains) showed significant changes, including 37 PUFA chains, 30 MUFA chains, and 2 SFA chains.

### 2.4. Lung, Brain, and Liver Showed Slight Lipidomic Alteration without Significant PUFA Change

We also examined the amount of PUFA chains in PC, PE, and TG in the lungs, brain, and liver within 5 min of irradiation and investigated the lipid changes in these organs. In the lung, the proportion of PUFA chains increased by 0.77% and 0.96% in PC, 0.35% and 0.27% in PE, and 1.10% and 2.19% in TG after 10 Gy and 20 Gy irradiation, respectively (Figure S2A,C,E). However, there were no significant changes in the intensity of total PUFA chains in lung PC, PE, and TG (Figure S2B,D,F).

Similarly, in the brain, the proportion of PUFA chains increased by 0.20% and 0.57% in PC, decreased by 0.22% and increased by 1.10% in PE, and decreased by 2.23% and 1.11% in TG under 10 Gy and 20 Gy irradiation within 5 min, respectively (Figure S3A,C,E). No significant changes were observed in the total PUFA chains in brain PC, PE, and TG (Figure S3B,D,F).

Regarding the liver, the percentage of PUFA chains in PC, PE, and TG decreased by 0.43% and increased by 0.25%, increased by 0.88% and 1.35%, and decreased by 2.30% and 1.75% (Figure S4A,C,E). However, there were no significant changes in the intensity of total PUFA chains in liver PC, PE, and TG (Figure S4B,D,F).

**Figure 4:**
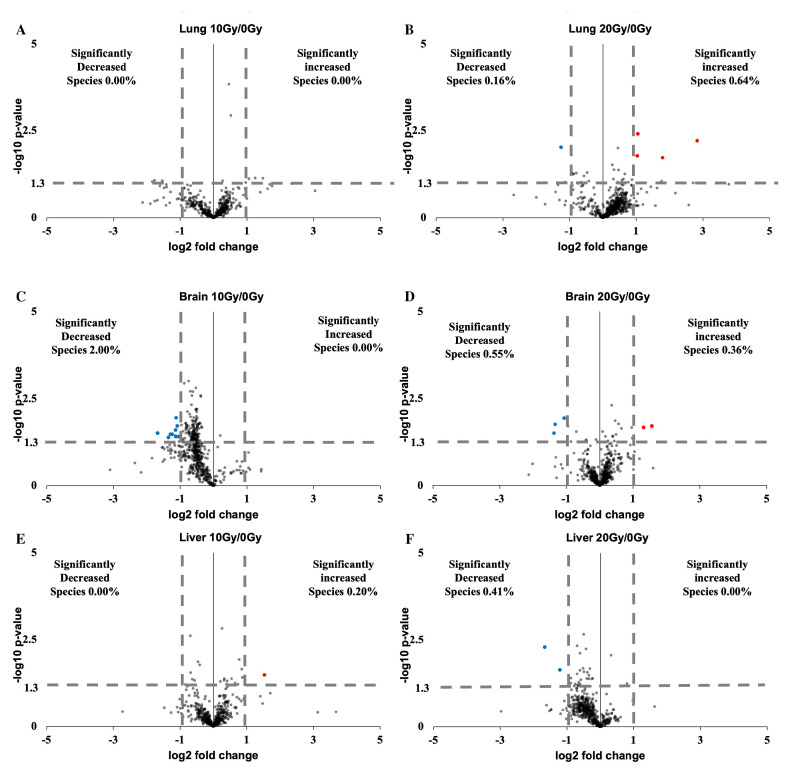
Lung, brain, and liver lipidomes within 5 min of irradiation. (**A**) Volcano plot of lipid profile under comparison between 10 Gy and 0 Gy irradiation in lung. (**B**) Volcano plot of lipid profile under comparison between 20 Gy and 0 Gy irradiation in lung. (**C**) Volcano plot of lipid profile under comparison between 10 Gy and 0 Gy irradiation in brain. (**D**) Volcano plot of lipid profile under comparison between 20 Gy and 0 Gy irradiation in brain. (**E**) Volcano plot of lipid profile under comparison between 10 Gy and 0 Gy irradiation in liver. (**F**) Volcano plot of lipid profile under comparison between 20 Gy and 0 Gy irradiation in liver.

Furthermore, we examined the lipidomic changes in the lung, brain, and liver within 5 min of radiation using volcano plots. Among the lung lipid group, 0.00% of the lipids showed a significant change after 10 Gy irradiation (Figure 4A). After 20 Gy irradiation, 0.64% of the lipids showed a significant increase. In comparison, 0.16% showed a significant decrease (Figure 4B). In the brain lipid group, 0% of the lipids showed a significant increase. In comparison, 2.00% showed a significant decrease after 10 Gy irradiation (Figure 4C). After 20 Gy irradiation, 0.36% of the lipids showed a significant increase. In comparison, 0.55% showed a significant decrease (Figure 4D). Among the liver lipid group, 0.2% of the lipids showed a significant increase. In comparison, 0% showed a significant decrease after 10 Gy irradiation (Figure 4E). After 20 Gy irradiation, 0% of the lipids showed a significant increase, and 0.41% showed a significant decrease (Figure 4F).

## 3. Discussion

In this research article, we present a novel LC-MS/MS analysis of fatty acid (FA) chains in the major lipid classes of mice’s multi-organ within a five-minute timeframe following irradiation. Our findings reveal a significant increase in PUFA chains of PC, PE, and TG specifically in the kidney after exposure to high-dose radiation. This increase in PUFA chains was not only an increase in the amount of PC, PE, and TG containing PUFA chains alone (i.e., a change in the number of homogeneous lipids), but also an increase in the proportion of PC-, PE-, and TG-containing PUFA chains (i.e., the appearance of new lipids containing PUFA chains). In other words, this implies that within a short period following high-dose radiation, the SFA and MUFA chains in the major kidney lipids are replaced by PUFA chains. Furthermore, this turnover of fatty acid chains is particularly pronounced in the significantly altered lipids of the kidney. Notably, we observe substantial changes in the kidney lipid group within the five-minute window after high-dose radiation, primarily characterized by growth.

Moreover, the growing lipids predominantly comprise PC, PE, and TG, with the most significant increases occurring in lipids containing PUFA chains. These findings suggest that the rapid impact of high-dose radiation on the lipidome primarily affects the fatty acid chain composition rather than the head group. In contrast to previous studies indicating a decrease in PUFA chains in phospholipids 72 h after irradiation [13], our study demonstrates an increase in PUFA chains within minutes following exposure to 10 Gy of radiation. This preference for elevated PUFA-containing lipids establishes the groundwork for free radical oxidation/peroxidation.

Considering that de novo lipogenesis exceeds a five-minute timeframe [16], we hypothesize that the replacement of the fatty acid chains described above occurs via the Land cycle [17]. The Land cycle has the potential for rapid large-scale reactions. Previous reports have shown that phospholipase A2 releases substantial amounts of arachidonic acid and lysolecithin from phospholipids within seconds [18,19]. In our study, we also examine the levels of lysophospholipids in the kidneys after irradiation, which serve as a source for phospholipid synthesis via the Land cycle. We observe a decrease in the levels of LPC and LPE following irradiation (Figure S5), indicating that the new lipids containing PUFA chains that emerge after irradiation may be synthesized from lysolecithin and PUFA. Based on the short-term fluctuations in lipid PUFA chain levels, we propose the PUFA protection hypothesis. This hypothesis suggests that PUFAs contain more double-bonded structures, which are more susceptible to ROS attacks. The substantial rise in PUFA chains within major lipids shortly after radiation implies an overall increased susceptibility of lipids to ROS. This increased susceptibility helps lipids compete with proteins or nucleic acids for ROS, thereby protecting proteins and nucleic acids from ROS-induced damage. Although the increase in PUFA chains promotes ROS attack on the plasma membrane, it represents the optimal cellular response (sacrificing lipids to protect proteins and nucleic acids) in the face of an acute or massive ROS attack.

It is essential to note that we employed two radiation doses, namely 10 Gy and 20 Gy. The PUFA chains in kidney lipids did not exhibit significant differences between the two doses. We attribute these findings to the limitations of short-term PUFA chain increases. We selected the organs primarily due to the brain’s and liver’s high lipid content, the kidney’s role as the main metabolic organ, and the lungs’ susceptibility to radiation damage. Contrary to our expectations, the PUFA chains in brain, liver, and lung lipids did not display significant changes within the first five minutes of irradiation. Theoretically, even in as little as five minutes, radiation-induced physicochemical reactions would occur in the lipids of all organs. However, due to the complex material composition within different organs, the extent of the biological reaction of lipids over a five-minute time frame will be different. One possible explanation for the observed organ-specific differences is the relationship between the intensity of very early reactions and the radiosensitivity of organ tissues [20,21]. Generally, lipid reactions are not typically considered when evaluating radiosensitivity. Nonetheless, our research highlights the importance of considering phenomena beyond those accounted for by the traditional linear-quadratic (LQ) model during the application of hypofractionated irradiation. To gain a more comprehensive understanding of the ultra-early lipid response to radiation in normal and tumor tissues, we will design relevant tumor-bearing mice, increase the time points, and increase the sample size in subsequent studies. These studies will help us to develop strategies to enhance the efficacy of tumor radiotherapy and mitigate the side effects of radiotherapy.

## 4. Materials and Methods

### 4.1. Animals Furthermore, X-ray Irradiation

Four-month-old C56BL/6 wild-type male mice (weight 26–28 g) were obtained from SLC Inc. (Hamamatsu, Japan). The mice were housed under a 12-h light/dark cycle for one week and provided unrestricted food and water access. Mice were randomly divided into three groups (n = 3 for each group). Group I mice were not irradiated and were used as the baseline group. Mice of groups II and III received whole-body irradiation with a single dose of 10 Gy and 20 Gy using an MX-160Labo X-ray machine (MediXtec, Nagoya, Japan), respectively. The dose rate was set to 1.5 Gy/min. After 5 min of X-ray radiation, all mice were dissected following cervical dislocation. Kidney, lung, brain, and liver tissue samples were collected rapidly and snap-frozen by dry ice. The frozen samples were then stored at −80 °C; until the lipid extraction.

### 4.2. Lipid Extraction

Lipids were extracted from kidney, lung, brain, and liver tissue by the modified Bligh and Dyer method [22]. The weight of the kidney was measured by a digital scale (PB3002-sdr, Mettler Toledo). Water was added at a rate of 8 µL/mg by weight of individual kidney, lung, brain, and liver samples. Then, a digital homogenizer was used to homogenize the samples with water (HK-1, AS ONE Corporation, Osaka, Japan). After homogenization, chloroform and methanol were added to the lysate. The mixture was transferred to a glass tube, mixed very well, and left at room temperature. After 10 min, extra chloroform was added and mixed very well. Then, 0.28 M acetic acid was added, mixed well, and centrifuged at 110× *g* for 15 min. The final ratio of water:methanol:chloroform:acetic acid was 1:2.5:2.5:1.25. An equal amount of the lower layer from each extract of a particular tissue was collected, followed by drying. The dried lipids were stored at −80 °C until LC-MS/MS analysis.

### 4.3. LC-MS and LC-MS/MS of Kidney, Lung, Brain, and Liver Lipids

The lipid was dissolved in 100% methanol containing 1 ng/µL PC (12:0/12:0) as internal standard. and transferred into a glass vial (Waters, New York, NY, USA) specified for LC-MS/MS with a glass insert (Systech, Scottsdale, AZ, USA) in it; a specific safe cap (Thermo Scientific, Lenexa, KS, USA) was screwed onto it for safe movement. Lipids were retrieved and analyzed by a Q Exactive™ Hybrid Quadrupole-Orbitrap™ mass spectrometer equipped with an electrospray ionization (ESI, Scottsdale, AZ, USA) source and attached to an Ultimate 3000 system (Thermo Scientific). The column and the sample temperature were maintained at 50 °C and 10 °C, respectively. A 10 µL lipid sample was injected into the machine and isolated in an Acclaim 120 C18 column (150 mm × 2.1 mm, 3 µm) (Thermo Scientific). The composition of the flow phase was depicted separately below by setting up the mobile phase as two parts, A and B. Part A consisted of water–acetonitrile–methanol (2:1:1 *v*/*v*/*v*), 5 mM ammonium formate, and 0.1% formic acid. Part B consisted of acetonitrile–isopropanol (1:9 *v*/*v*), 5 mM ammonium formate, and 0.1% formic acid. The elution flow rate was programmed at 300 µL/min using a series of linear-gradient beginning with 20% solvent B, increasing linearly to 100% B within 50 min, sustaining 100% B for 60 min, then reducing linearly to 20% B between 60 min and 60.1 min, and terminating with 20% B within the final 10 min. The total operation time was 70 min, and conditions of the MS instruments were optimized as follows: probe heater temperature, 350 °C; S-lens RF level, 50; capillary temperature, 250 °C; auxiliary gas flow, 15 (au); sheath gas flow, 50 (au); sweep gas flow, 0 (au); spray voltage in positive mode, 3.5 kV, and negative mode, 2.5 kV. Data were acquired from 2.5 min to 60 min in both full MS and data-dependent MS/MS (dd-MS2) modes. The parameters of full MS mode were set as follows: automatic gain control (AGC) target, $1\times 10^{6}$; mass resolving power, 70,000 (FWHM, at *m*/*z* 200); recorded *m*/*z* range, 220–2000; and maximum injection time (IT), 100 ms. For dd-MS2, the following conditions were used: mass resolving power, 17,500 (FWHM, at *m*/*z* 200); AGC target, $1\times 10^{5}$; maximum IT, 80 ms; loop count, 5; TopN, 5; isolation window, 2.0 *m*/*z*; normalized collision energy (NCE), 30.0 eV; stepped NCE, 15.0% for positive mode and 35.0% for negative mode, and dynamic exclusion, 15.0 s.

### 4.4. Lipid Identification Furthermore, Quantification

We acquired the spectral data with the Xcalibur v3.0 Software (Thermo Scientific). The data was then subjected to LipidSearch™ software version 4.2.13 (Mitsui Knowledge Industry, Tokyo, Japan) for lipid identification and quantification [23,24,25]. The lipids were identified as lipid ions. We set the following parameters: database as HCD; retention time as 0.01 min; search type as product_QEX; precursor tolerance as 5.0 ppm; and product tolerance as 8.0 ppm. Identification quality filters of A, B, and C were applied. We used 0.01 *m*/*z* tolerance and −1.0 min to 2.0 min of the retention time range for the quantification. After identification, alignment was performed with a 0.25 retention time tolerance.

### 4.5. Data Processing

Lipid intensities recorded in the Xcalibur v3.0 software and area values of lipid species identified by LipidSearch™ software were divided by the area values of the internal standard PC(12:0_12:0) for normalization.

### 4.6. Data Analysis

After acquiring the annotated and quantitated data of lipidomic tissue, we separated them into different groups following the experimental conditions and managed to make comparisons to seek the variations between those groups. We used MS Excel to create pie charts and volcano plots with the normalized data. We divide the average of the various lipid intensities to obtain fold change. We then applied Student’s *t*-test to examine the significance of the lipid alteration. We considered the lipids that showed an alteration with a fold change more significant than 2 and a *p*-value smaller than or equal to 0.05 as significantly altered. Then we used a scatter plot and set log2 fold change as X and −log10 *p*-value as Y to build the volcano plots.

We found that the lipids displayed significant statistical differences, and the accuracy of the auto-annotation of LipidSearch was confirmed using Xcalibur (Thermo fisher scientific) (Figure S6).

GraphPad produced the bar graphs (Prism version.8.0.2.263).

## 5. Conclusions

Our findings indicate that the kidneys of mice exhibit a significant increase in PUFA chains within 5 min of exposure to 10 Gy and 20 Gy radiation. However, no significant changes in PUFA chains were observed in the lung, brain, and liver lipids. In the kidney, PC’s and PE’s main PUFA chain components were C18:2, C20:4, and C22:6, which exhibited a significant increase. Similarly, the major PUFA chain component in kidney TG, C18:2, significantly rose. Additionally, we observed significant changes in the lipidome of the kidney within 5 min of radiation, characterized by an overall increase in lipids. These findings shed light on the acute biochemical response of multiple organs to high-dose radiation, as elucidated from a lipidomic perspective.

## Data Availability

All relevant data are within the paper and its Supporting Information files.

## Supplementary Materials

The following supporting information can be downloaded at: https://www.mdpi.com/article/10.3390/ijms241512439/s1.
