# Supplementary material for: Lipid Polyunsaturated Fatty Acid Chains in Mouse Kidneys Were Increased within 5 min of a Single High Dose Whole Body Irradiation
Source: Int J Mol Sci. 2023 Aug 4;24(15):12439. doi: 10.3390/ijms241512439 (PMC10419980; doi:10.3390/ijms241512439)
Supplement: Supplementary file 1 [file ijms-24-12439-s001.zip › Supplementary Figures.docx]

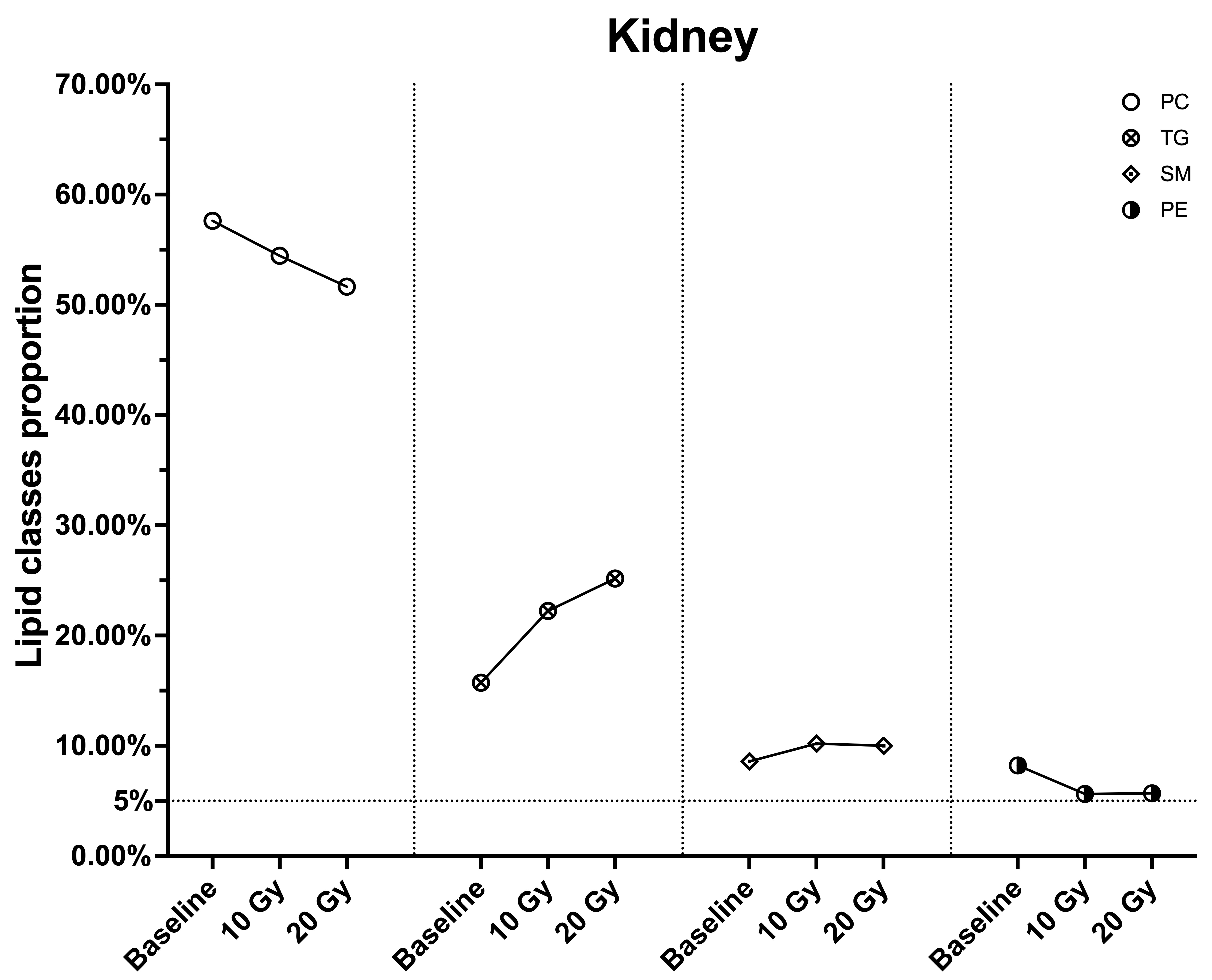


Figure S1: Proportion of PC, PE, TG, and SM lipid class in the whole lipids of kidney.


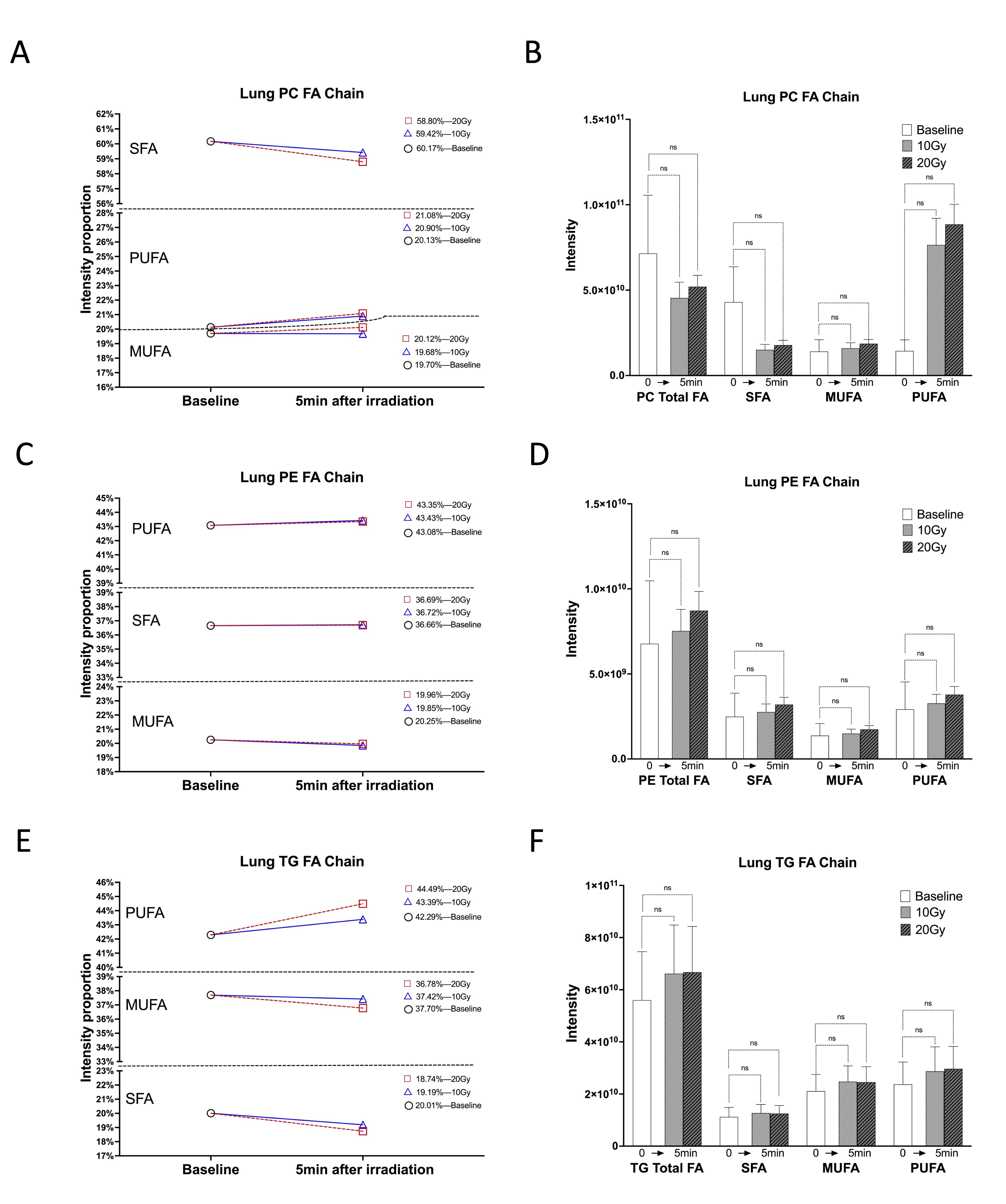


Figure S2: FA chains of lung lipids barely changed under 10 Gy and 20 Gy irradiation in 5 min.


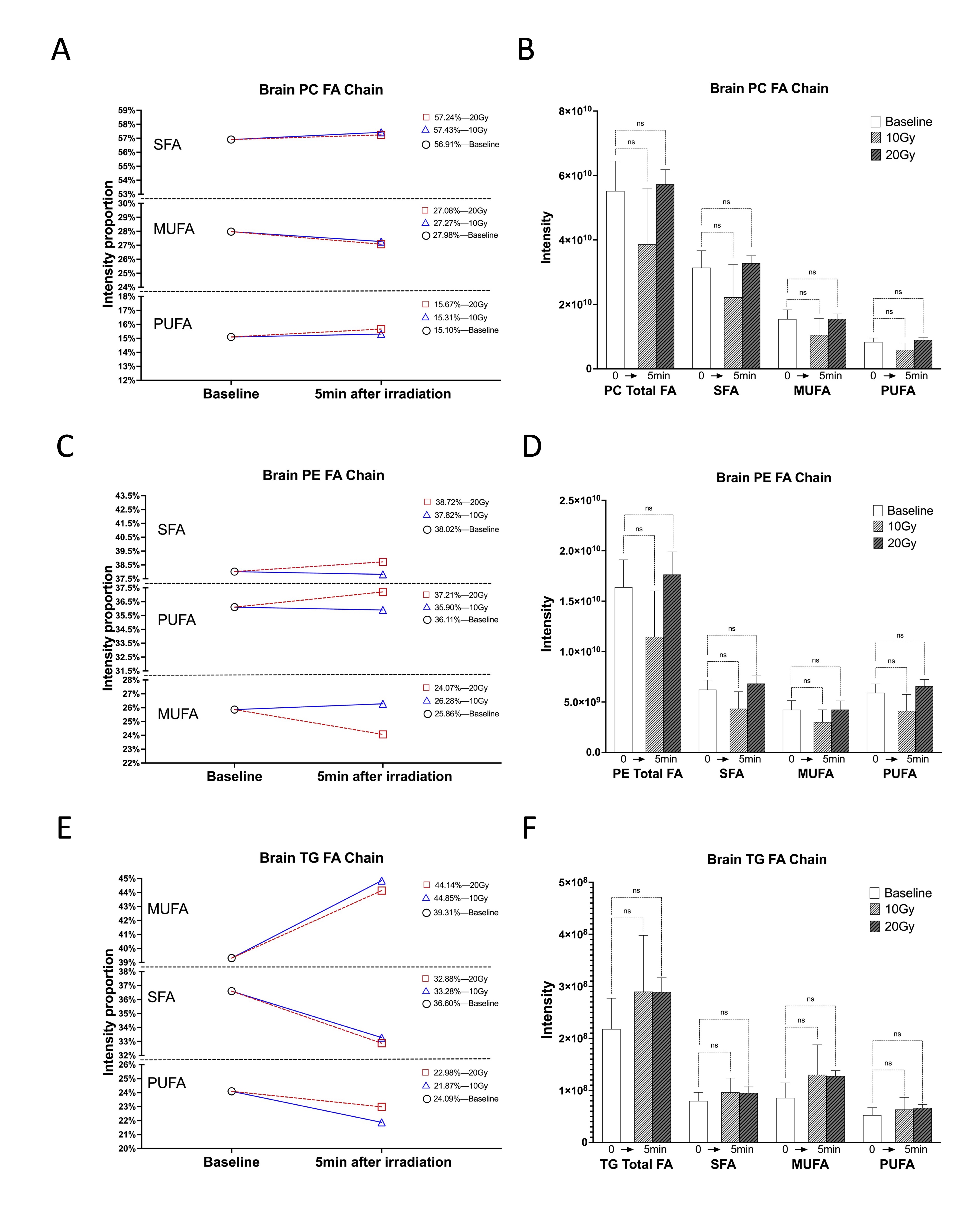


Figure S3: FA chains of brain lipids barely changed under 10 Gy and 20 Gy irradiation in 5 min.


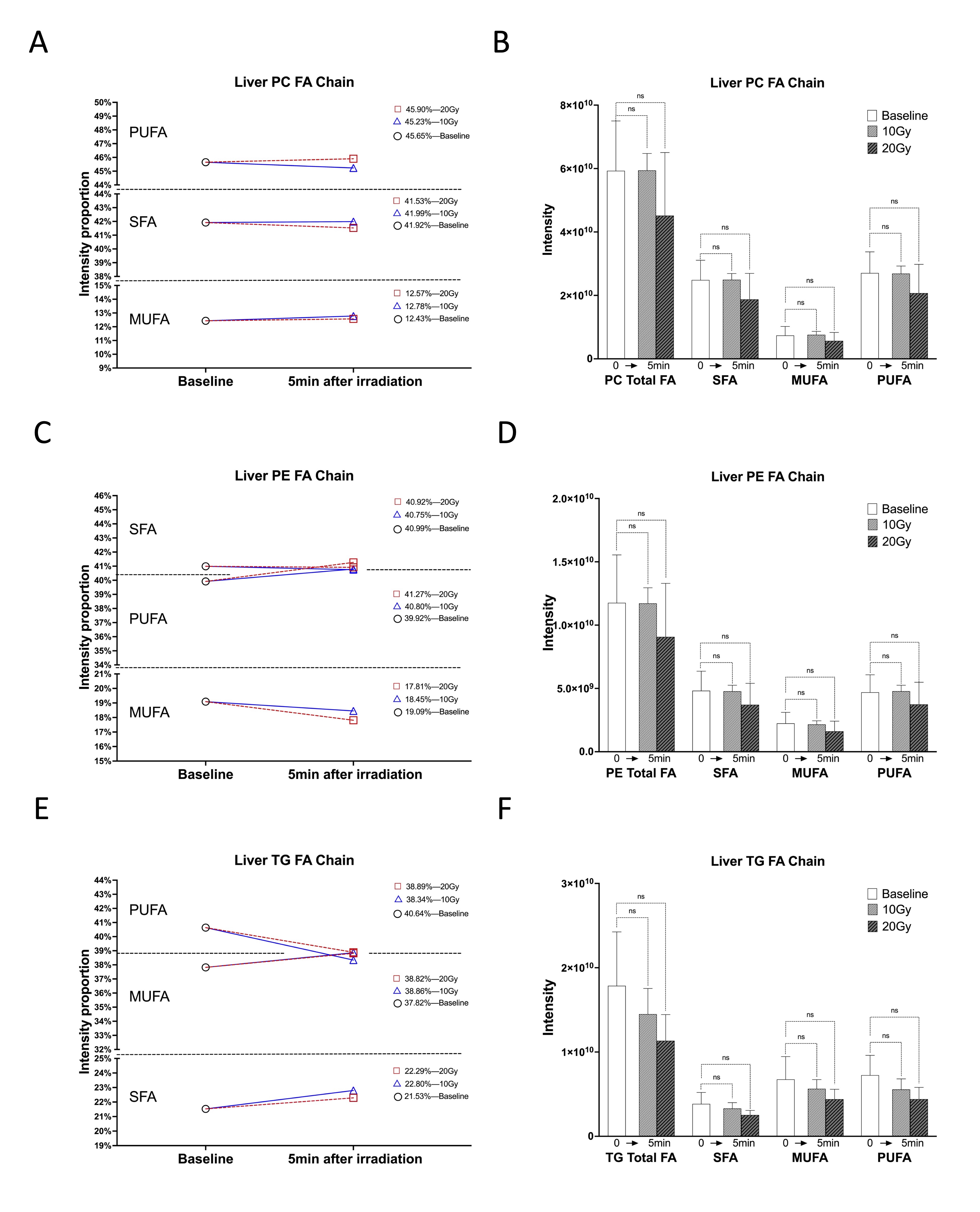


Figure S4: FA chains of liver lipids barely changed under 10 Gy and 20 Gy irradiation in 5 min.


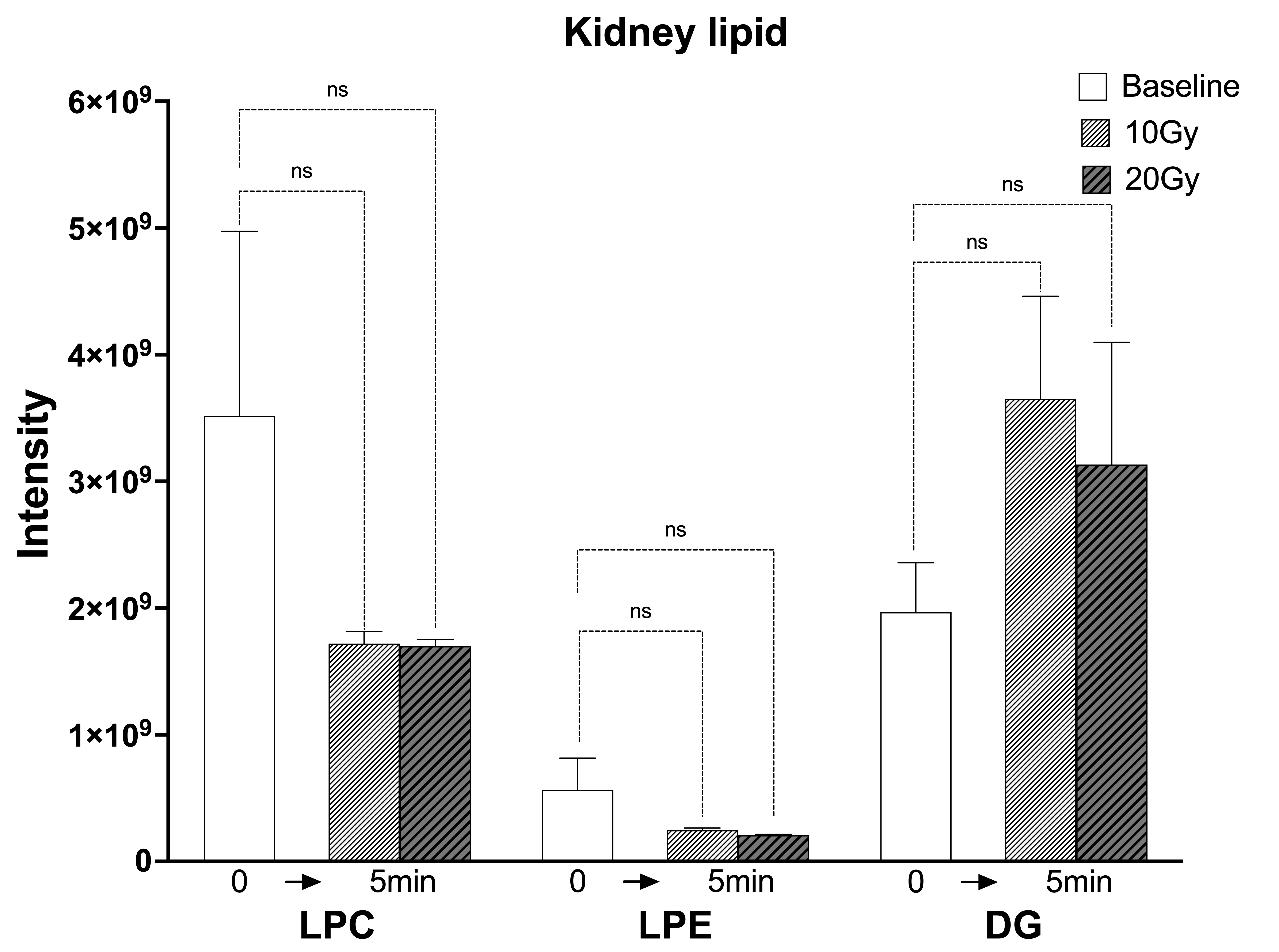


Figure S5: Intensity histograms of kidney LPC, LPE, and DG under 0 Gy, 10 Gy, and 20 Gy irradiation.





Figure S6: MS/MS spectrum and illustration for annotations of lipids.

Table S1: Lipidomic data of kidney, lung, brain, and liver;

Table S2: Composition of fatty acids chains in PC, PE, and TG.
