# Supplementary material for: Lipid Polyunsaturated Fatty Acid Chains in Mouse Kidneys Were Increased within 5 min of a Single High Dose Whole Body Irradiation
Source: Int J Mol Sci. 2023 Aug 4;24(15):12439. doi: 10.3390/ijms241512439 (PMC10419980; doi:10.3390/ijms241512439)
Supplement: Supplementary file 1 [file ijms-24-12439-s001.zip › Table S2.pdf]

Table S2: Composition of fatty acids chains in PC, PE, and TG.

lipid	SFA					MUFA					PUFA				
	Normalized abundance (Mean±SD)			FC		Normalized abundance (Mean±SD)			FC		Normalized abundance (Mean±SD)			FC	
	0Gy	10Gy	20Gy	10Gy/0 Gy	20Gy/0 Gy	0Gy	10Gy	20Gy	10Gy/0 Gy	20Gy/0 Gy	0Gy	10Gy	20Gy	10Gy/0 Gy	20Gy/0 Gy
PC	9.1±2.5	11.0±1.2	10.4±0.6	1.21	1.14	2.3±0.6	2.6±0.5	2.5±0.2	1.14	1.08	6.5±1.4	9.6±1.4	9.1±0.5	1.49*	1.41
PE	11.2±3.6	17.3±2.0	16.9±1.1	1.55*	1.51	5.9±1.5	9.6±1.2	9.3±0.6	1.63*	1.58	9.6±2.5	16.7±1.8	16.4±1.0	1.74*	1.71
TG	1.2±0.8	2.1±0.4	2.4±0.8	1.72	1.97	4.2±3.2	8.0±1.7	9.0±3.5	1.90	2.13	1.9±1.5	4.2±0.9	4.7±1.2	2.20	2.48*

Mean, mean value; SD, standard deviation; FC, folds change; *, p-value≤0.05
